# Regulation of Leukocytes by TspanC8 Tetraspanins and the “Molecular Scissor” ADAM10

**DOI:** 10.3389/fimmu.2018.01451

**Published:** 2018-07-02

**Authors:** Alexandra L. Matthews, Chek Ziu Koo, Justyna Szyroka, Neale Harrison, Aditi Kanhere, Michael G. Tomlinson

**Affiliations:** ^1^School of Biosciences, College of Life and Environmental Sciences, University of Birmingham, Birmingham, United Kingdom; ^2^Centre of Membrane Proteins and Receptors (COMPARE), University of Birmingham, Birmingham, United Kingdom

**Keywords:** a disintegrin and metalloproteinase 10, tetraspanins, TspanC8s, leukocytes

## Abstract

A disintegrin and metalloproteinase 10 (ADAM10) is a ubiquitous transmembrane protein that functions as a “molecular scissor” to cleave the extracellular regions from its transmembrane target proteins. ADAM10 is well characterized as the ligand-dependent activator of Notch proteins, which control cell fate decisions. Indeed, conditional knockouts of ADAM10 in mice reveal impaired B-, T-, and myeloid cell development and/or function. ADAM10 cleaves many other leukocyte-expressed substrates. On B-cells, ADAM10 cleavage of the low-affinity IgE receptor CD23 promotes allergy and asthma, cleavage of ICOS ligand impairs antibody responses, and cleavage of the BAFF–APRIL receptor transmembrane activator and CAML interactor, and BAFF receptor, reduce B-cell survival. On microglia, increased ADAM10 cleavage of a rare variant of the scavenger receptor triggering receptor expressed on myeloid cells 2 may increase susceptibility to Alzheimer’s disease. We and others recently showed that ADAM10 interacts with one of six different regulatory tetraspanin membrane proteins, which we termed the TspanC8 subgroup, comprising Tspan5, Tspan10, Tspan14, Tspan15, Tspan17, and Tspan33. The TspanC8s are required for ADAM10 exit from the endoplasmic reticulum, and emerging evidence suggests that they dictate ADAM10 subcellular localization and substrate specificity. Therefore, we propose that ADAM10 should not be regarded as a single scissor, but as six different scissors with distinct substrate specificities, depending on the associated TspanC8. In this review, we collate recent transcriptomic data to present the TspanC8 repertoires of leukocytes, and we discuss the potential role of the six TspanC8/ADAM10 scissors in leukocyte development and function.

## Introduction

The proteolytic cleavage, or “shedding,” of the extracellular region (ectodomain) of transmembrane proteins is an important mechanism for the regulation of leukocyte development and function. Shedding can initiate intracellular signal transduction *via* the cell-associated cleavage fragment (e.g., Notch signaling to drive cell fate decisions), downregulate signaling or adhesion that requires cell surface receptor expression, or activate paracrine signaling through the release of growth factors, cytokines, and chemokines (1). The ADAMs (a disintegrin and metalloproteinases) are one of the main proteinase families that function as sheddases and can be regarded as “molecular scissors.” A total of 22 ADAM genes have been identified in humans, of which 12 (ADAM8, 9, 10, 12, 15, 17, 19, 20, 21, 28, 30, and 33) are active proteases with the consensus sequence (HExGHxxGxxHD) required for Zn^2+^-dependent protease activity (1).

## The “Molecular Scissor” ADAM10

**Figure 1:**
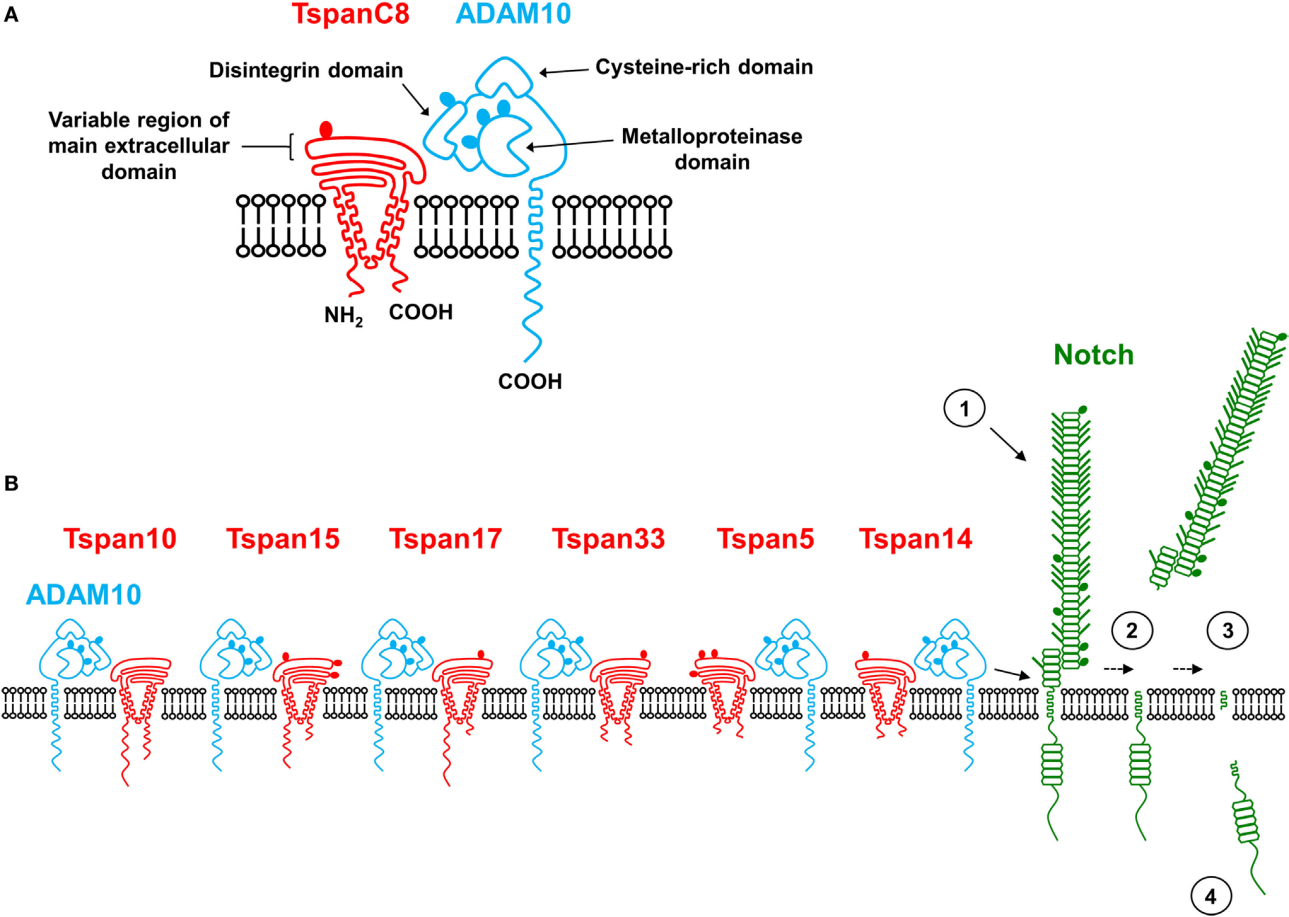
Six ADAM10 scissors: Tspan14 and Tspan5 may be important for Notch activation in immune cells. **(A)** A schematic representation of a TspanC8 and ADAM10. **(B)** A model figure to show the six different TspanC8/ADAM10 complexes, which have different subcellular localizations, distinct substrate specificities, and may have different ADAM10 conformations. Notch cleavage is initiated following engagement with a Notch ligand on another cell (1), which induces a conformational change that we hypothesize allows cleavage by a Tspan14/ADAM10 or Tspan5/ADAM10 scissor (2), followed by intramembrane cleavage by γ-secretase (3), to release the intracellular domain to act as a transcription factor and drive cell fate decisions (4). Tspan10 has also been implicated in Notch activation, but its relatively low expression by immune cells suggests no substantial role in these cells. N-linked and O-linked glycosylation sites are indicated by filled ovals and short lines, respectively.

One of the best-characterized ADAMs is ADAM10, due to its essential role in ligand-dependent cleavage of Notch proteins to initiate Notch signaling (2). Indeed, ADAM10-knockout mice die at embryonic day 9.5, phenocopying double knockout mice for two of the four Notch proteins, Notch 1 and 4 (3, 4). ADAM10 has an N-terminal signal sequence, an inhibitory prodomain, a metalloproteinase domain, followed by disintegrin, cysteine-rich, transmembrane, and cytoplasmic domains (Figure 1). The prodomain is removed by proprotein convertases during biosynthesis to generate a mature sheddase (5). The first crystal structure of the mature ADAM10 ectodomain suggests that the metalloprotease exists in a closed conformation in which the cysteine-rich domain partially occludes the catalytic site, but with the catalytic site in position to cleave substrates close to the plasma membrane (6). ADAM10 has at least 40 substrates, including amyloid precursor protein (7), cellular prion protein (8), cadherins (9–11), and the platelet-activating collagen/fibrin receptor GPVI (12, 13). ADAM10 has a number of substrates that are expressed by leukocytes, or which impact on leukocyte function, including the low-affinity IgE receptor CD23 (14, 15), the endothelial cell–cell adhesion molecule vascular-endothelial (VE)-cadherin (11), the B-cell costimulatory molecule ICOS ligand (16), B-cell homeostasis proteins transmembrane activator and CAML interactor (TACI) (17) and BAFF receptor (BAFFR) (18), and triggering receptor expressed on myeloid cells 2 (TREM2) (19, 20). ADAM10 has been implicated in myriad immune diseases including T-cell acute lymphoblastic leukemia (T-ALL), asthma, atherosclerosis, and Alzheimer’s disease (2).

## Tetraspanins as “Membrane Organizers”

The tetraspanins are a superfamily of transmembrane proteins expressed in multicellular eukaryotes. They are characterized by four transmembrane domains and small and large extracellular loops (Figure 1), the latter containing structurally important cysteine residues. Mammals express 33 tetraspanins and each cell type has a distinct repertoire of tetraspanins (21); leukocytes express at least 20 tetraspanins (22). Tetraspanins are “membrane organizers” that form dynamic nanoclusters (21). Visualization of tetraspanins CD37, CD53, CD81, and CD82 on human B-cells and dendritic cells, by super-resolution microscopy, suggests that approximately 10 tetraspanins of a single type cluster together into individual nanodomains (23). Tetraspanins also associate directly with specific non-tetraspanin proteins, termed “partner proteins,” to regulate their intracellular trafficking, clustering, lateral mobility, and compartmentalization (21). Relatively well-studied tetraspanin–partner interactions are tetraspanin CD151 with the laminin-binding integrins (24), tetraspanin CD81 with the B-cell co-receptor CD19 (25), and Tspan12 with the Wnt/Norrin receptor Frizzled-4 (26). In each case, tetraspanin mutations yield phenotypes that are consistent with impaired partner protein function. For example, in CD81-deficient humans and mice, CD19 fails to traffic to the B-cell surface and antibody generation is impaired (25). The recently published crystal structure of full-length CD81, which is the first such structure for a tetraspanin, shows CD81 to be cone shaped with an intramembrane cholesterol-binding cavity within the transmembranes (27). Molecular dynamics simulations suggest that CD81 may exist in two different conformations, a closed conformation when cholesterol is present, and an open conformation when cholesterol is absent, in which the large extracellular loop swings upwards (27). Therefore, tetraspanins could function as “molecular switches” that control the activity of their partner proteins through cholesterol-regulated conformational change.

## TspanC8 Tetraspanins Regulate ADAM10: The “Six Scissor” Hypothesis

In 2012, we and others showed that ADAM10 interacts with six tetraspanins that are closely related by protein sequence: Tspan5, Tspan10, Tspan14, Tspan15, Tspan17, and Tspan33 (28–30). We termed these the TspanC8 subgroup due to the eight cysteine residues in their large extracellular loops (28, 29); other tetraspanins have four, six, or seven cysteines. The TspanC8s are essential for promoting ADAM10 exit from the endoplasmic reticulum, its subsequent maturation in the Golgi through removal of the prodomain, and trafficking to the cell surface or other membrane compartments (28–30). The functional association between TspanC8s and ADAM10 has been demonstrated in TspanC8-knockout mice (29, 31) and the fruit fly *Drosophila* (28), and is reinforced by recent data demonstrating reciprocal regulation of Tspan5 exit from the endoplasmic reticulum by ADAM10 (32). Moreover, emerging evidence indicates that each TspanC8 can target ADAM10 to different subcellular localizations and to different substrates, and ADAM10 may adopt distinct conformations dictated by the associated TspanC8 (28, 32–35). For example, we and two other groups reported Tspan15 as the only TspanC8 to promote ADAM10 cleavage of neuronal (N)-cadherin (30, 33, 34). These *in vitro* data are supported by data from the recently characterized Tspan15-deficient mouse, which has strikingly reduced N-cadherin cleavage in the brain, despite only a subtle decrease in mature ADAM10 expression (31). ADAM10 shedding of Notch is promoted by Tspan5, Tspan10, and Tspan14 (Figure 1), but not by Tspan15 and 33 (28, 32, 33, 36). In addition, we have shown Tspan5 and Tspan17 to regulate VE-cadherin expression on endothelial cells (35). Taking these data together, we now propose that ADAM10 should be regarded as six different TspanC8/ADAM10 scissor complexes, rather than a single scissor (37, 38). This idea has implications for therapeutic targeting of ADAM10, which may be impractical due to toxic side effects. However, targeting one of the TspanC8/ADAM10 complexes, *via* the tetraspanin, may minimize toxicity while enabling substrate- and disease-specific targeting.

We and others have recently reviewed our current understanding of how TspanC8s regulate ADAM10 (37–39). In this review, we will analyze and present published RNA-Seq transcriptomic data for TspanC8 expression in different leukocyte subsets. We will discuss these expression profiles in the context of our current knowledge of ADAM10’s role in the development and function of T-cells, B-cells, and myeloid cells.

## Regulation of T-Cell Development and Transmigration by ADAM10 and TspanC8s

Two publications have shown that ADAM10 is important for normal T-cell development, most likely through regulation of Notch signaling (40, 41). In the first, the embryonic lethality of ADAM10-knockout mice was circumvented by the generation of transgenic mice that express dominant negative ADAM10 under the control of the T-cell-specific Lck promoter (40). The dominant negative ADAM10 construct yields a similar phenotype to T-cell-specific deletion of Notch1, the Notch family member with the major role in thymocyte development. Thymocyte numbers are reduced by 60–90% due to a partial block in the CD4/CD8 double negative to double positive transition, with accompanying reduction in expression of T-cell receptor (TCR) β (40). Defective Notch signaling is the probable mechanism, since expression of Notch-responsive genes is partially reduced, and partial rescue is achieved by transgenic overexpression of the Notch ligand Delta-1, or a dominant active form of Notch1, in thymocytes. The dominant negative construct lacks the metalloproteinase domain and is expressed at several-fold higher levels than endogenous ADAM10 (40); one mechanism of action may be the sequestration of endogenous TspanC8s, since we have shown such a construct to interact with TspanC8s (34). In the second publication, conditional T-cell-specific ADAM10-knockout mice were generated by crossing ADAM10 floxed mice with mice expressing Cre recombinase driven by the Lck promoter (41). This model phenocopies T-cell-specific Notch1 deletion in showing a twofold to threefold reduction in thymocyte numbers, due to a partial block in development from the double negative to double positive stage. There is reduced expression of Notch target genes but, unlike the dominant negative ADAM10 model, no defect in TCRβ expression is observed (41).

**Figure 2:**
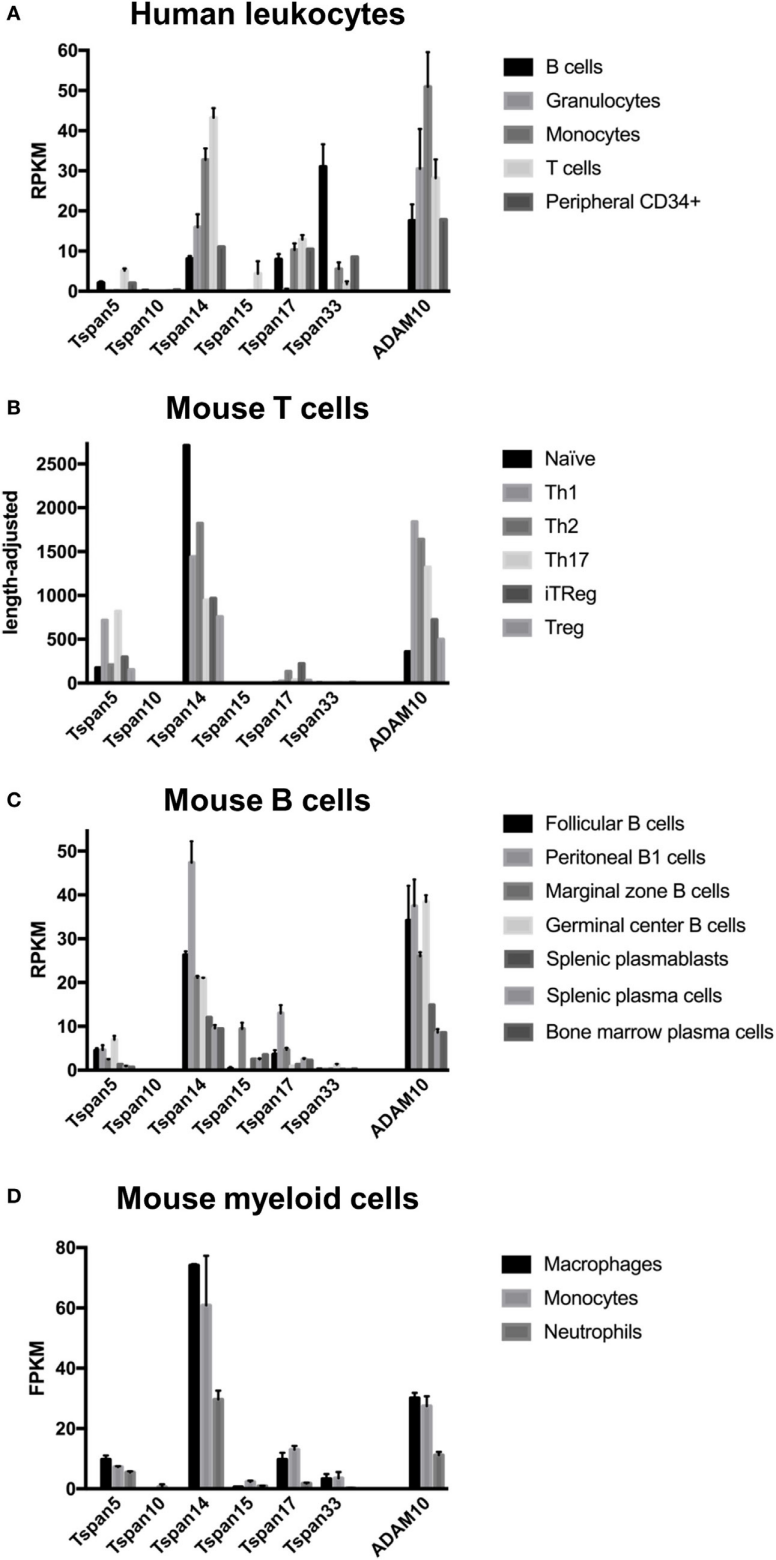
Leukocytes express ADAM10, but different cell subsets have distinct TspanC8 repertoires. Publically available RNA-Seq data for **(A)** human leukocytes [Gene Expression Omnibus (GEO) accession GSE51984], **(B)** mouse T-cell subsets (43), **(C)** mouse B-cells (GEO accession GSE60927), and **(D)** mouse macrophages, monocytes, and neutrophils (GEO accession GSE59831). Data are presented as reads per kilobase of transcript per million mapped reads (RPKM) **(A,C)**, as length-adjusted values that provide a measure equivalent to RPKM (43) **(B)**, or as fragments per kilobase of transcript per million mapped reads (FPKM) **(D)**. Error bars represent the SD. Number of samples are as follows: five for panel **(A)**, except for CD34+ peripheral cells (hematopoietic stem cells from the blood) which has one; two for panel **(C)**, except for splenic plasmablasts and bone marrow plasma cells which have one, and splenic plasma cells which have three; and two for panel **(D)**, with the exception of neutrophils which have two.

The most highly expressed TspanC8 in human and mouse T-cells is Tspan14, followed by Tspan5 and Tspan17; Tspan15 is also expressed by human T-cells but not mouse (Figures 2A,B). However, it is important to note that such publically available transcriptomic data have not been independently validated, nor have the expression profiles been confirmed using validated antibodies. Nevertheless, since both Tspan14 and Tspan5 promote Notch signaling (28, 32, 33), we hypothesize that Tspan14/ADAM10 will have a major role in thymocyte development *via* activation of Notch1, while Tspan5/ADAM10 may have a minor role. The future analyses of the respective knockout mice will help to test this hypothesis; the Tspan14-knockout mouse has yet to be made, while the Tspan5-knockout mouse is viable but functionally uncharacterized (32). It is possible that Tspan14 and/or Tspan5 play a role in the aggressive blood cancer T-ALL. Approximately 50% of T-ALL is driven by activating mutations in Notch1, some of which require ADAM10 for full activation in a ligand-independent manner. Knockdown of ADAM10 reduces Notch signaling and T-ALL proliferation (42). Targeting Tspan14 or Tspan5 may achieve a similar result, yet without the toxicity of global ADAM10 inhibition. Interestingly, antibody targeting of Tspan5 can impair Notch signaling (32).

In addition to Notch proteins, a number of other proteins have been reported to be cleaved by ADAM10 on T-cells: CD40 ligand (44), Fas ligand (45, 46), LAG-3 (47), CD44 (48), and T-cell immunoglobulin and mucin domain 3 (Tim-3) (49). How important these cleavage events are to T-cell function has yet to be determined, and for CD40 ligand, LAG-3 and Tim-3 is complicated by their additional cleavage by ADAM17 (44, 47, 49), an ADAM10-related metalloproteinase.

We have recently reported that endothelial cell-expressed ADAM10 promotes the transmigration (also known as extravasation or diapedesis) of T-cells in an *in vitro* model of inflammation (35). The mechanism involves ADAM10 regulation of VE-cadherin expression levels, since ADAM10 knockdown results in 50% elevated VE-cadherin expression and delayed transmigration, which is rescued by partial VE-cadherin knockdown to wild-type levels. This promotion of transmigration by endothelial ADAM10 appears restricted to T-cells, since no effects on neutrophils or B-cell transmigration are observed (35), nor on monocytes in a separate study (50). Among the TspanC8s, we have previously reported that Tspan14 is the most highly expressed on endothelial cells (29, 51). However, knockdown experiments show that Tspan5 and Tspan17 are the tetraspanins that promote ADAM10 regulation of VE-cadherin (35). Consistent with this common function, Tspan5 and Tspan17 are the most highly conserved pair of tetraspanins, with 72% protein sequence identity in human (35). However, it remains to be determined whether this role for Tspan5/17 holds true *in vivo*. Tspan17-knockout mice have yet to be made; ultimately a Tspan5/17 double knockout may be required to overcome functional redundancy.

## Regulation of B-Cell Development and Function by ADAM10 and Potential Role for TspanC8s

An important role for ADAM10 in B-cell development and function has been demonstrated using B-cell-specific ADAM10-knockout mice, made by crossing ADAM10 floxed mice with CD19-Cre mice (52). Early development of B-cells in the bone marrow of these mice is unaltered, with normal numbers of pro-, pre-, and immature B-cell populations. B1 cell numbers in the peritoneal cavity are also normal. However, ADAM10-knockout immature B-cells entering the spleen fail to develop into marginal zone B-cells; these cells act as a first line of defense by rapid antibody generation against blood-borne pathogens that become trapped in the spleen. By contrast, follicular zone B-cell numbers in the spleen are slightly elevated. The mechanism underlying defective marginal zone B-cell development in the absence of ADAM10 appears to be defective Notch2 activation (52), and consistent with this, B-cells express relatively high levels of Notch2 and low levels of Notch 1, 3, and 4, and Notch2 is critical for development of marginal zone but not follicular B-cells (53–55).

B-cell-specific ADAM10-knockout mice have a striking reduction in antibody responses following immunization, associated with impaired germinal center formation in secondary lymphoid tissues (56). The mechanism responsible appears to be the upregulated expression of TNFα, a pro-inflammatory cytokine which is important for the maintenance of lymphoid tissue architecture, and ADAM17, which sheds TNFα (57). How ADAM10-deficiency leads to ADAM17 and TNFα upregulation in B-cells remains unknown. To investigate the function of ADAM10-knockout plasma cells in the context of normal germinal centers, ADAM10 floxed mice were crossed with IgG1-Cre mice to delete ADAM10 post-isotype switching. Antibody responses are strikingly reduced, despite normal plasma cell numbers (58). This may be due to elevated expression of ICOS ligand, a recently identified ADAM10 substrate, on ADAM10-knockout B-cells (16). The engagement of ICOS ligand with its receptor ICOS on T-cells is required for T-cell-dependent antibody responses. In ADAM10-knockout B-cells, elevated ICOSL causes a substantial reduction in surface ICOS by promoting its internalization (16), thus providing a mechanism for impaired antibody responses.

With the possible exception of Notch2, the best-studied ADAM10 substrate on B-cells is CD23, the low-affinity IgE receptor, which regulates allergic and inflammatory responses (14, 15). Indeed, on ADAM10-knockout B-cells, CD23 expression is increased approximately threefold, while soluble CD23 levels in plasma are substantially reduced (52). In an IgE-dependent asthma model, B-cell-specific ADAM10-knockout mice have strikingly reduced signs of allergic inflammation in the lung (59). Allergic patients and allergy-prone Th2 mice have increased expression of ADAM10 on B-cells and increased soluble CD23 and IgE levels in plasma (60). Although the regulation of IgE expression by CD23 is complex and not fully understood, these data have lead the authors to propose ADAM10 as a therapeutic target for asthma (59).

ADAM10 is emerging as a regulator of the BAFF–APRIL system which controls B-cell homeostasis. The two ligands are BAFF and APRIL, while the three receptors are B-cell maturation antigen, TACI, and BAFFR, the latter of which binds BAFF but not APRIL. ADAM10 can shed TACI to release a soluble ectodomain that acts as a decoy receptor, binding to BAFF and APRIL and so inhibiting survival of B-cells (17). In addition, cell survival can be reduced by ADAM10 or ADAM17 shedding of BAFFR (18).

In human B-cells, Tspan33 expression is highest, followed by Tspan14, Tspan17, and then Tspan5 (Figure 2A). In mouse, Tspan14 expression is highest, followed by Tspan5, Tspan15, and Tspan17, while Tspan33 expression is minimal (Figure 2C). Therefore, Tspan14 is likely to be the main regulator of Notch2 in B-cells, with a less important role for Tspan5. A role for TspanC8s in regulating CD23 shedding has not been reported, but Tspan5, Tspan14, Tspan17, and/or Tspan33 are candidates; this is important work for the future, because such a tetraspanin is a potential therapeutic target for asthma. Tspan33 expression in human B-cells has been confirmed at the protein level (61), but the ADAM10 substrates that it regulates have not been investigated.

## Regulation of Myeloid Cell Function by ADAM10 and Potential Role for TspanC8s

Dendritic cell-specific ADAM10-knockout mice have been generated by crossing ADAM10 floxed mice with CD11c-Cre mice (62). These mice have strikingly impaired Th2 responses, but Th1 and Th17 responses are unaffected. As a consequence, the mice are protected from IgE-mediated anaphylaxis and allergic lung inflammation. This appears to be due to defective Notch signaling, since rescue is observed by transgenic expression of the Notch1 intracellular domain, and dendritic cell-specific Notch1-knockout mice have a similar phenotype (62). Our RNA-Seq analyses have found Tspan14 to be most highly expressed in human dendritic cells, with lower expression of Tspan17 and Tspan33 (data not shown). Therefore, Tspan14 is likely to be important in dendritic cells for Notch signaling.

Specific knockout of ADAM10 in myeloid cells has been investigated by crossing ADAM10 floxed mice with LysM-cre mice (63). This does not achieve complete knockout in myeloid cells, but surface levels of ADAM10 on bone marrow-derived macrophages are reduced by 85%. No major abnormalities in the mice are observed, and leukocyte populations are present in normal numbers. However, ADAM10-knockout results in a reduced inflammatory phenotype in macrophages and a reduced capacity to migrate and to degrade extracellular matrix (63). Macrophages play a central role in the initiation and progression of the inflammatory disease atherosclerosis, and can take up lipids to become pro-inflammatory foam cells within atherosclerotic plaques. To investigate the role of macrophage ADAM10 in this disease, bone marrow from myeloid-specific ADAM10-knockout mice was transplanted into atherosclerosis-prone low-density lipoprotein receptor knockout mice. Consistent with a less inflammatory macrophage phenotype, atherosclerotic plaques appear more stable, with higher collagen content, although plaque size is similar to wild type (63). Nevertheless, this has important implications for human disease; stable plaques are less susceptible to the rupture that causes thrombosis, vessel occlusion, and heart attack or stroke. Interestingly, Notch signaling promotes an inflammatory macrophage phenotype, and blockade of Notch signaling in an atherosclerosis model reduces atherosclerosis development while increasing plaque stability (64). Macrophages express relatively high levels of the Notch-promoting Tspan14 (Figures 2A,D), highlighting the macrophage Tspan14/ADAM10 complex as a potential therapeutic target for maintaining plaque stability in atherosclerosis.

An additional study generated mice with leukocyte- and myeloid-specific ADAM10 deficiency by crossing floxed ADAM10 mice with Vav-Cre and LysM-Cre mice, respectively. In an inflammatory lung model, neutrophil and monocyte recruitment are reduced by approximately 50% in the absence of ADAM10 (65). The underlying mechanism is not clear. Tspan14 appears to be the only TspanC8 expressed by human granulocytes and is also highly expressed by mouse neutrophils, which have relatively weak Tspan5 expression (Figures 2A,D). Therefore, Tspan14/ADAM10-induced Notch activation may potentially promote neutrophil inflammatory responses.

Finally, the scavenger receptor TREM2 has been recently identified as an ADAM10 substrate with a potential role in Alzheimer’s disease (19, 20). TREM2 is expressed on macrophages, microglia, osteoclasts, and dendritic cells. A rare H157Y variant of TREM2 is associated with increased risk of Alzheimer’s disease; amino acid 157 is at the cleavage site for ADAM10, and H157Y is shed more readily. The loss of TREM2 renders macrophages less phagocytic, and the authors propose that this renders the individual more prone to Alzheimer’s disease (19, 20). It will be interesting to determine if any macrophage-expressed TspanC8, namely Tspan5, Tspan14, Tspan17, or Tspan33, can promote TREM2 cleavage, and could thus be considered a therapeutic target for TREM2-associated Alzheimer’s disease.

## Concluding Remarks

The six scissor hypothesis suggests that ADAM10 should be studied in the context of its regulatory tetraspanins. In leukocytes, the relatively high expression of Tspan14, together with its capacity to promote Notch activation, suggest that the Tspan14/ADAM10 complex may be critical for leukocyte development and function. The future analyses of cells and mice deficient in Tspan14, and other TspanC8s, will determine which scissor cleaves which substrates. This may direct therapeutic targeting of individual TspanC8/ADAM10 complexes, using antibodies or small molecules, to modulate a specific substrate while avoiding the toxicity of global ADAM10 targeting. Such an approach could provide new treatments for ADAM10-associated diseases, including T-ALL, asthma, atherosclerosis, and Alzheimer’s disease.

## Author Contributions

AM, CK, JS, NH, and MT: conception, design, and writing of the manuscript. AK: data analysis and editing of the manuscript.

## Conflict of Interest Statement

The authors declare that the research was conducted in the absence of any commercial or financial relationships that could be construed as a potential conflict of interest.

## References

[1] Giebeler N Zigrino P. A disintegrin and metalloprotease (ADAM): historical overview of their functions. Toxins (Basel) (2016) 8:122. 10.3390/toxins8040122 27120619 PMC4848645

[2] Wetzel S Seipold L Saftig P. The metalloproteinase ADAM10: a useful therapeutic target? Biochim Biophys Acta (2017) 1864(11 Pt B):2071–81. 10.1016/j.bbamcr.2017.06.005 28624438

[3] Hartmann D de Strooper B Serneels L Craessaerts K Herreman A Annaert W The disintegrin/metalloprotease ADAM 10 is essential for Notch signalling but not for alpha-secretase activity in fibroblasts. Hum Mol Genet (2002) 11:2615–24. 10.1093/hmg/11.21.2615 12354787

[4] Krebs LT Xue Y Norton CR Shutter JR Maguire M Sundberg JP Notch signaling is essential for vascular morphogenesis in mice. Genes Dev (2000) 14:1343–52. 10.1101/gad.14.11.1343 10837027 PMC316662

[5] Wong E Maretzky T Peleg Y Blobel CP Sagi I. The functional maturation of a disintegrin and metalloproteinase (ADAM) 9, 10, and 17 requires processing at a newly identified proprotein convertase (PC) cleavage site. J Biol Chem (2015) 290:12135–46. 10.1074/jbc.M114.624072 25795784 PMC4424348

[6] Seegar TCM Killingsworth LB Saha N Meyer PA Patra D Zimmerman B Structural basis for regulated proteolysis by the alpha-secretase ADAM10. Cell (2017) 171:1638–48.e7. 10.1016/j.cell.2017.11.014 29224781 PMC5773094

[7] Postina R Schroeder A Dewachter I Bohl J Schmitt U Kojro E A disintegrin-metalloproteinase prevents amyloid plaque formation and hippocampal defects in an Alzheimer disease mouse model. J Clin Invest (2004) 113:1456–64. 10.1172/JCI20864 15146243 PMC406531

[8] Altmeppen HC Prox J Krasemann S Puig B Kruszewski K Dohler F The sheddase ADAM10 is a potent modulator of prion disease. Elife (2015) 4. 10.7554/eLife.04260 25654651 PMC4346534

[9] Maretzky T Reiss K Ludwig A Buchholz J Scholz F Proksch E ADAM10 mediates E-cadherin shedding and regulates epithelial cell-cell adhesion, migration, and beta-catenin translocation. Proc Natl Acad Sci U S A (2005) 102:9182–7. 10.1073/pnas.0500918102 15958533 PMC1166595

[10] Reiss K Maretzky T Ludwig A Tousseyn T de Strooper B Hartmann D ADAM10 cleavage of N-cadherin and regulation of cell-cell adhesion and beta-catenin nuclear signalling. EMBO J (2005) 24:742–52. 10.1038/sj.emboj.7600548 15692570 PMC549617

[11] Schulz B Pruessmeyer J Maretzky T Ludwig A Blobel CP Saftig P ADAM10 regulates endothelial permeability and T-cell transmigration by proteolysis of vascular endothelial cadherin. Circ Res (2008) 102:1192–201. 10.1161/CIRCRESAHA.107.169805 18420943 PMC2818019

[12] Bender M Hofmann S Stegner D Chalaris A Bosl M Braun A Differentially regulated GPVI ectodomain shedding by multiple platelet-expressed proteinases. Blood (2010) 116:3347–55. 10.1182/blood-2010-06-289108 20644114

[13] Gardiner EE Karunakaran D Shen Y Arthur JF Andrews RK Berndt MC. Controlled shedding of platelet glycoprotein (GP)VI and GPIb-IX-V by ADAM family metalloproteinases. J Thromb Haemost (2007) 5:1530–7. 10.1111/j.1538-7836.2007.02590.x 17445093

[14] Lemieux GA Blumenkron F Yeung N Zhou P Williams J Grammer AC The low affinity IgE receptor (CD23) is cleaved by the metalloproteinase ADAM10. J Biol Chem (2007) 282:14836–44. 10.1074/jbc.M608414200 17389606 PMC2582392

[15] Weskamp G Ford JW Sturgill J Martin S Docherty AJ Swendeman S ADAM10 is a principal ‘sheddase’ of the low-affinity immunoglobulin E receptor CD23. Nat Immunol (2006) 7:1293–8. 10.1038/ni1399 17072319

[16] Lownik JC Luker AJ Damle SR Cooley LF El Sayed R Hutloff A ADAM10-mediated ICOS ligand shedding on B cells is necessary for proper T cell ICOS regulation and T follicular helper responses. J Immunol (2017) 199:2305–15. 10.4049/jimmunol.1700833 28814605 PMC5605448

[17] Hoffmann FS Kuhn PH Laurent SA Hauck SM Berer K Wendlinger SA The immunoregulator soluble TACI is released by ADAM10 and reflects B cell activation in autoimmunity. J Immunol (2015) 194:542–52. 10.4049/jimmunol.1402070 25505277 PMC4282951

[18] Smulski CR Kury P Seidel LM Staiger HS Edinger AK Willen L BAFF- and TACI-dependent processing of BAFFR by ADAM proteases regulates the survival of B cells. Cell Rep (2017) 18:2189–202. 10.1016/j.celrep.2017.02.005 28249164

[19] Schlepckow K Kleinberger G Fukumori A Feederle R Lichtenthaler SF Steiner H An Alzheimer-associated TREM2 variant occurs at the ADAM cleavage site and affects shedding and phagocytic function. EMBO Mol Med (2017) 9:1356–65. 10.15252/emmm.201707672 28855300 PMC5623859

[20] Thornton P Sevalle J Deery MJ Fraser G Zhou Y Stahl S TREM2 shedding by cleavage at the H157-S158 bond is accelerated for the Alzheimer’s disease-associated H157Y variant. EMBO Mol Med (2017) 9:1366–78. 10.15252/emmm.201707673 28855301 PMC5623839

[21] Charrin S Jouannet S Boucheix C Rubinstein E. Tetraspanins at a glance. J Cell Sci (2014) 127:3641–8. 10.1242/jcs.154906 25128561

[22] Tarrant JM Robb L van Spriel AB Wright MD. Tetraspanins: molecular organisers of the leukocyte surface. Trends Immunol (2003) 24:610–7. 10.1016/j.it.2003.09.011 14596886

[23] Zuidscherwoude M Gottfert F Dunlock VM Figdor CG van den Bogaart G van Spriel AB. The tetraspanin web revisited by super-resolution microscopy. Sci Rep (2015) 5:12201. 10.1038/srep12201 26183063 PMC4505338

[24] Hemler ME. Tetraspanin proteins promote multiple cancer stages. Nat Rev Cancer (2014) 14:49–60. 10.1038/nrc3640 24505619

[25] Levy S. Function of the tetraspanin molecule CD81 in B and T cells. Immunol Res (2014) 58:179–85. 10.1007/s12026-014-8490-7 24522698

[26] Tomlinson MG Eye-opening potential for tetraspanin Tspan12 as a therapeutic target for diseases of the retinal vasculature. Circulation (2017) 136:196–9. 10.1161/CIRCULATIONAHA.117.028521 28696267

[27] Zimmerman B Kelly B McMillan BJ Seegar TC Dror RO Kruse AC Crystal structure of a full-length human tetraspanin reveals a cholesterol-binding pocket. Cell (2016) 167:1041–51. 10.1016/j.cell.2016.09.056 27881302 PMC5127602

[28] Dornier E Coumailleau F Ottavi JF Moretti J Boucheix C Mauduit P TspanC8 tetraspanins regulate ADAM10/Kuzbanian trafficking and promote Notch activation in flies and mammals. J Cell Biol (2012) 199:481–96. 10.1083/jcb.201201133 23091066 PMC3483123

[29] Haining EJ Yang J Bailey RL Khan K Collier R Tsai S The TspanC8 subgroup of tetraspanins interacts with A disintegrin and metalloprotease 10 (ADAM10) and regulates its maturation and cell surface expression. J Biol Chem (2012) 287:39753–65. 10.1074/jbc.M112.416503 23035126 PMC3501075

[30] Prox J Willenbrock M Weber S Lehmann T Schmidt-Arras D Schwanbeck R Tetraspanin15 regulates cellular trafficking and activity of the ectodomain sheddase ADAM10. Cell Mol Life Sci (2012) 69:2919–32. 10.1007/s00018-012-0960-2 22446748 PMC11114675

[31] Seipold L Altmeppen H Koudelka T Tholey A Kasparek P Sedlacek R In vivo regulation of the A disintegrin and metalloproteinase 10 (ADAM10) by the tetraspanin 15. Cell Mol Life Sci (2018) 1–17. 10.1007/s00018-018-2791-2 29520422 PMC11105247

[32] Saint-Pol J Billard M Dornier E Eschenbrenner E Danglot L Boucheix C New insights into the tetraspanin Tspan5 using novel monoclonal antibodies. J Biol Chem (2017) 292:9551–66. 10.1074/jbc.M116.765669 28428248 PMC5465482

[33] Jouannet S Saint-Pol J Fernandez L Nguyen V Charrin S Boucheix C TspanC8 tetraspanins differentially regulate the cleavage of ADAM10 substrates, Notch activation and ADAM10 membrane compartmentalization. Cell Mol Life Sci (2016) 73:1895–915. 10.1007/s00018-015-2111-z 26686862 PMC4819958

[34] Noy PJ Yang J Reyat JS Matthews AL Charlton AE Furmston J TspanC8 tetraspanins and A disintegrin and metalloprotease 10 (ADAM10) interact via their extracellular regions: evidence for distinct binding mechanisms for different TspanC8 proteins. J Biol Chem (2016) 291:3145–57. 10.1074/jbc.M115.703058 26668317 PMC4751363

[35] Reyat JS Chimen M Noy PJ Szyroka J Rainger GE Tomlinson MG. ADAM10-Interacting tetraspanins Tspan5 and Tspan17 regulate VE-cadherin expression and promote T lymphocyte transmigration. J Immunol (2017) 199:666–76. 10.4049/jimmunol.1600713 28600292 PMC5502317

[36] Zhou J Fujiwara T Ye S Li X Zhao H. Downregulation of Notch modulators, tetraspanin 5 and 10, inhibits osteoclastogenesis in vitro. Calcif Tissue Int (2014) 95:209–17. 10.1007/s00223-014-9883-2 24935633 PMC4139439

[37] Matthews AL Noy PJ Reyat JS Tomlinson MG. Regulation of A disintegrin and metalloproteinase (ADAM) family sheddases ADAM10 and ADAM17: the emerging role of tetraspanins and rhomboids. Platelets (2017) 28:333–41. 10.1080/09537104.2016.1184751 27256961 PMC5490636

[38] Matthews AL Szyroka J Collier R Noy PJ Tomlinson MG. Scissor sisters: regulation of ADAM10 by the TspanC8 tetraspanins. Biochem Soc Trans (2017) 45:719–30. 10.1042/BST20160290 28620033 PMC5473022

[39] Saint-Pol J Eschenbrenner E Dornier E Boucheix C Charrin S Rubinstein E. Regulation of the trafficking and the function of the metalloprotease ADAM10 by tetraspanins. Biochem Soc Trans (2017) 45:937–44. 10.1042/BST20160296 28687716

[40] Manilay JO Anderson AC Kang C Robey EA. Impairment of thymocyte development by dominant-negative Kuzbanian (ADAM-10) is rescued by the Notch ligand, delta-1. J Immunol (2005) 174:6732–41. 10.4049/jimmunol.174.11.6732 15905513

[41] Tian L Wu X Chi C Han M Xu T Zhuang Y. ADAM10 is essential for proteolytic activation of Notch during thymocyte development. Int Immunol (2008) 20:1181–7. 10.1093/intimm/dxn076 18635581

[42] Sulis ML Saftig P Ferrando AA. Redundancy and specificity of the metalloprotease system mediating oncogenic NOTCH1 activation in T-ALL. Leukemia (2011) 25:1564–9. 10.1038/leu.2011.130 21625236 PMC3165074

[43] Stubbington MJ Mahata B Svensson V Deonarine A Nissen JK Betz AG An atlas of mouse CD4(+) T cell transcriptomes. Biol Direct (2015) 10:14. 10.1186/s13062-015-0045-x 25886751 PMC4384382

[44] Yacoub D Benslimane N Al-Zoobi L Hassan G Nadiri A Mourad W. CD154 is released from T-cells by A disintegrin and metalloproteinase domain-containing protein 10 (ADAM10) and ADAM17 in a CD40 protein-dependent manner. J Biol Chem (2013) 288:36083–93. 10.1074/jbc.M113.506220 24189063 PMC3861656

[45] Kirkin V Cahuzac N Guardiola-Serrano F Huault S Luckerath K Friedmann E The Fas ligand intracellular domain is released by ADAM10 and SPPL2a cleavage in T-cells. Cell Death Differ (2007) 14:1678–87. 10.1038/sj.cdd.4402175 17557115

[46] Schulte M Reiss K Lettau M Maretzky T Ludwig A Hartmann D ADAM10 regulates FasL cell surface expression and modulates FasL-induced cytotoxicity and activation-induced cell death. Cell Death Differ (2007) 14:1040–9. 10.1038/sj.cdd.4402101 17290285

[47] Li N Wang Y Forbes K Vignali KM Heale BS Saftig P Metalloproteases regulate T-cell proliferation and effector function via LAG-3. EMBO J (2007) 26:494–504. 10.1038/sj.emboj.7601520 17245433 PMC1783452

[48] Anderegg U Eichenberg T Parthaune T Haiduk C Saalbach A Milkova L ADAM10 is the constitutive functional sheddase of CD44 in human melanoma cells. J Invest Dermatol (2009) 129:1471–82. 10.1038/jid.2008.323 18971959

[49] Moller-Hackbarth K Dewitz C Schweigert O Trad A Garbers C Rose-John S and metalloprotease (ADAM) 10 and ADAM17 are major sheddases of T cell immunoglobulin and mucin domain 3 (Tim-3). J Biol Chem (2013) 288:34529–44. 10.1074/jbc.M113.488478 24121505 PMC3843067

[50] Tsubota Y Frey JM Tai PW Welikson RE Raines EW. Monocyte ADAM17 promotes diapedesis during transendothelial migration: identification of steps and substrates targeted by metalloproteinases. J Immunol (2013) 190:4236–44. 10.4049/jimmunol.1300046 23479224 PMC3622190

[51] Bailey RL Herbert JM Khan K Heath VL Bicknell R Tomlinson MG. The emerging role of tetraspanin microdomains on endothelial cells. Biochem Soc Trans (2011) 39:1667–73. 10.1042/BST20110745 22103505

[52] Gibb DR El Shikh M Kang DJ Rowe WJ El Sayed R Cichy J ADAM10 is essential for Notch2-dependent marginal zone B cell development and CD23 cleavage in vivo. J Exp Med (2010) 207:623–35. 10.1084/jem.20091990 20156974 PMC2839139

[53] Moriyama Y Sekine C Koyanagi A Koyama N Ogata H Chiba S Delta-like 1 is essential for the maintenance of marginal zone B cells in normal mice but not in autoimmune mice. Int Immunol (2008) 20:763–73. 10.1093/intimm/dxn034 18381350

[54] Saito T Chiba S Ichikawa M Kunisato A Asai T Shimizu K Notch2 is preferentially expressed in mature B cells and indispensable for marginal zone B lineage development. Immunity (2003) 18:675–85. 10.1016/S1074-7613(03)00111-0 12753744

[55] Santos MA Sarmento LM Rebelo M Doce AA Maillard I Dumortier A Notch1 engagement by delta-like-1 promotes differentiation of B lymphocytes to antibody-secreting cells. Proc Natl Acad Sci U S A (2007) 104:15454–9. 10.1073/pnas.0702891104 17878313 PMC2000509

[56] Chaimowitz NS Martin RK Cichy J Gibb DR Patil P Kang DJ A disintegrin and metalloproteinase 10 regulates antibody production and maintenance of lymphoid architecture. J Immunol (2011) 187:5114–22. 10.4049/jimmunol.1102172 21998451 PMC4006936

[57] Folgosa L Zellner HB El Shikh ME Conrad DH Disturbed follicular architecture in B cell A disintegrin and metalloproteinase (ADAM)10 knockouts is mediated by compensatory increases in ADAM17 and TNF-alpha shedding. J Immunol (2013) 191:5951–8. 10.4049/jimmunol.1302042 24227779 PMC3863601

[58] Chaimowitz NS Kang DJ Dean LM Conrad DH. ADAM10 regulates transcription factor expression required for plasma cell function. PLoS One (2012) 7:e42694. 10.1371/journal.pone.0042694 22880085 PMC3411801

[59] Mathews JA Ford J Norton S Kang D Dellinger A Gibb DR A potential new target for asthma therapy: a disintegrin and metalloprotease 10 (ADAM10) involvement in murine experimental asthma. Allergy (2011) 66:1193–200. 10.1111/j.1398-9995.2011.02614.x 21557750 PMC3963393

[60] Cooley LF Martin RK Zellner HB Irani AM Uram-Tuculescu C El Shikh ME Increased B Cell ADAM10 in allergic patients and Th2 prone mice. PLoS One (2015) 10:e0124331. 10.1371/journal.pone.0124331 25933166 PMC4416757

[61] Perez-Martinez CA Maravillas-Montero JL Meza-Herrera I Vences-Catalan F Zlotnik A Santos-Argumedo L. Tspan33 is expressed in transitional and memory B cells, but is not responsible for high ADAM10 expression. Scand J Immunol (2017) 86:23–30. 10.1111/sji.12559 28449222

[62] Damle SR Martin RK Cockburn CL Lownik JC Carlyon JA Smith AD ADAM10 and Notch1 on murine dendritic cells control the development of type 2 immunity and IgE production. Allergy (2018) 73:125–36. 10.1111/all.13261 28745029 PMC5739941

[63] van der Vorst EP Jeurissen M Wolfs IM Keijbeck A Theodorou K Wijnands E Myeloid A disintegrin and metalloproteinase domain 10 deficiency modulates atherosclerotic plaque composition by shifting the balance from inflammation toward fibrosis. Am J Pathol (2015) 185:1145–55. 10.1016/j.ajpath.2014.11.028 25659879

[64] Fukuda D Aikawa E Swirski FK Novobrantseva TI Kotelianski V Gorgun CZ Notch ligand delta-like 4 blockade attenuates atherosclerosis and metabolic disorders. Proc Natl Acad Sci U S A (2012) 109:E1868–77. 10.1073/pnas.1116889109 22699504 PMC3390871

[65] Pruessmeyer J Hess FM Alert H Groth E Pasqualon T Schwarz N Leukocytes require ADAM10 but not ADAM17 for their migration and inflammatory recruitment into the alveolar space. Blood (2014) 123:4077–88. 10.1182/blood-2013-09-511543 24833351

